# Chemical Profiles of Cultivated Agarwood Induced by Different Techniques

**DOI:** 10.3390/molecules24101990

**Published:** 2019-05-24

**Authors:** Tingting Yan, Sheng Yang, Yuan Chen, Qian Wang, Gaiyun Li

**Affiliations:** 1 Research Institute of Forestry New Technology, Chinese Academy of Forestry, Beijing 100091, China; yantt@criwi.org.cn; 2 Research Institute of Wood Industry, Chinese Academy of Forestry, Beijing 100091, China; yangsheng@criwi.org.cn (S.Y.); chenyuan@criwi.org.cn (Y.C.); 15501130520@163.com (Q.W.)

**Keywords:** cultivated agarwood, *Aquilaria sinensis*, 2-(2-phenylethyl)-chromone, sesquiterpenoid

## Abstract

Agarwood is the resinous wood produced in some *Aquilaria* species and is highly valued for wide usages in medicine, incense, and perfume. To protect the threatened *Aquilaria* species, the cultivation of *Aquilaria sinensis* and artificial agarwood induction techniques have been effectively established in China. To evaluate the quality of agarwood induced by different techniques, patterns of chemical constituents in artificial agarwood by four methods (wounding using an axe, burning-chisel-drilling, chemical inducer, and biological inoculation) were analyzed and compared by UPLC-ESI-MS/MS and GC-EI-MS in this study. Results of GC-MS gave a panorama of chemical constituents in agarwood, including aromatic compounds, steroids, fatty acids, sesquiterpenoids, and 2-(2-phenlyethyl)-chromones (PECs). Sesquiterpenoids were dominant in agarwood induced by wounding using an axe. PEC comprised over 60% of components in agarwood produced by biological inoculation and chemical inducers. PECs were identified by UPLC-ESI-MS/MS in all artificial agarwood and the relative contents varied in different groups. Tetrahydro-2-(2-phenylethyl)-chromones (THPECs) in wounding by axes induced agarwood were lower while 2-(2-phenylethyl)-chromones (FPECs) were higher than other groups. The results showed that methods used for inducing agarwood formation in *Aquilaria sinensis* affect the chemical constituents of agarwood.

## 1. Introduction

The process of agarwood formation is initiated in the parenchyma cells in some species belonging to *Thymelaeaceae* family [1]. The cells activate protective machinery to synthesize secondary metabolites which defend against the stress [2]. The chemicals are produced, stored, and distributed in the wounded areas, filling the anatomical compartments [3]. Based on the process, various artificial agarwood induction methods have been invented and classified into two groups: conventional and nonconventional methods [4]. The conventional techniques include physical intrusions such as burning-chisel-drilling and wounding using an axe, which have been established for nearly 1000 years in ancient China [5]. Non-conventional methods have been newly developed in recent years [4]. Solvents containing ions or some microbes are injected into trunks of health trees to stimulate agarwood formation [6,7]. These methods are intensively practiced in plantations of China and other agarwood producing countries [5]. Agarwood produced by artificial induction techniques will inevitably be the majority in the commercial and medical market in the near future. Therefore, the quality of cultivated agarwood is of great concern. Some recent reports suggested that agarwood induced by nonconventional methods were comparable with wild agarwood while others reported the obvious differences in chemical constituents between wild and cultivated agarwood [8,9,10]. Studies focusing on agarwood yielded by conventional induction methods are still few [11]. Analysis and comparison of chemical constituents in agarwood induced by different methods is obligatory to provide comprehensive assessments of them.

More and more researchers are beginning to engage in illustrating the mechanisms behind secondary metabolites synthesis during agarwood formation for better performance of artificial agarwood techniques [4,12,13]. Chromones are detectable during the first 20 days after treatment of chemical inducers [14]. Major chromones will all appear and proportions become steady after nine months [15]. The results suggested formation of agarwood is complex and dynamic. Whether induction methods affect chemical constituents in agarwood is still unknown. The conventional induction methods somehow simulate the formation procedure of wild agarwood which is produced by physical wounding caused by heavy wind blowing, lightning striking, or insects biting [5]. However, procedures for agarwood formation by conventional methods are more time-consuming and laborious [16]. Non-conventional techniques are reported with high yielding within one-year induction but persistent accumulation of secondary metabolites inside trees are difficult [17]. Detailed comparisons on chemical constituents of artificial agarwood might also help us to know whether agarwood induction methods affect the components in resins, which will aidthe improvement of artificial induction techniques.

Chemical constituents in agarwood have been intensively studied by multiple analysis methods in recent years [1]. Main classes of aromatic constituents in agarwood are 2-(2-phenylethyl)chromones (PECs) [18]. Research conducted mainly through LC-MS/MS and NMR analysis showed chromones are grouped into four types by their backbone structures: tetrahydro-2-(2-phenylethyl)chromones (THPECs), epoxy-(2-phenylethyl)chromones (EPECs), diepoxy-(2-phenylethyl)chromones (DEPECs), and PECs [11]. Relative contents of chromones in agarwood were reported to change during agarwood formation [7]. Some reports suggested certain 2-(2-phenylethyl)chromones accumulated in high-quality agarwood [19,20,21]. Other reports mainly through GC-MS or NMR provide evidences for specific sesquiterpenes in agarwood of higher prices in the market [22,23]. Accumulative studies showed both chromones and sesquiterpenes are crucial for the medical and fragrance usages [18,24,25]. Therefore, chemical profiles of both chromones and sesquiterpenes are necessary for qualification of agarwood. In order to depict chemical profiles in artificial agarwood, LC-MS/MS and GC-MS combined with metabolomics were applied to compare the chemical characters of artificial agarwood in this study.

Agarwood samples separately induced by two conventional methods and two non-conventional methods were used in this study. Chemical constituents were analyzed by GC-MS and LC-MS/MS for the purpose of revealing differencs of chemical profiles in agarwood induced by different techniques. The detected compounds by GC-MS were identified by searching NIST14 or matching with reference compounds. Qualitative analysis of 2-(2-phenylethyl)chromones were conducted by UPLC-MS/MS based on previous reports. All raw data from GC-MS and LC-MS were preprocessed and further used for multivariated analysis as reported in metabolomics studies. The results strongly suggested induction methods affect chemical profiles in artifical agarwood.

## 2. Results

### 2.1. Morphological Observations of Agarwoods Induced by Different Techniques

**Figure 1:**
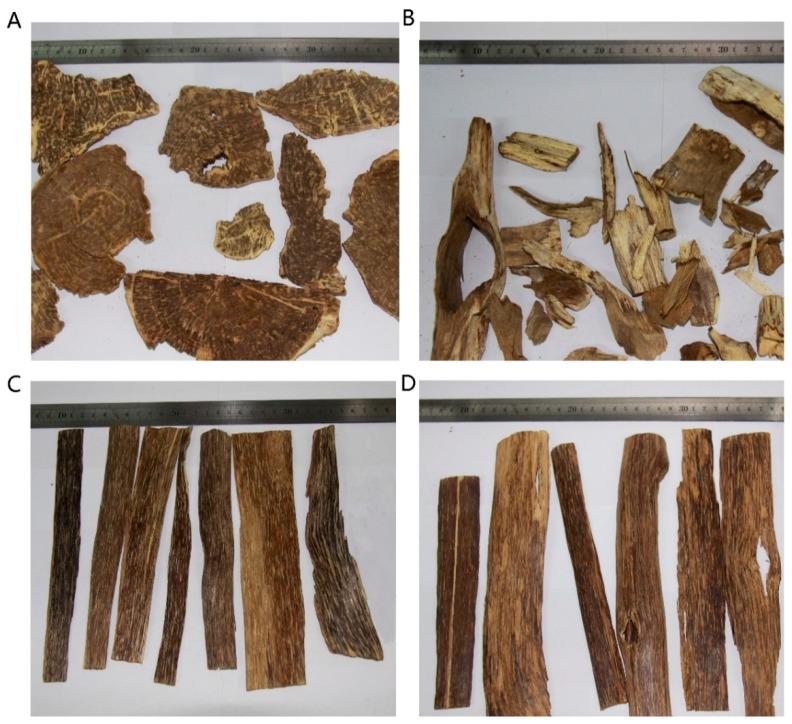
Morphological observations of artificial agarwood by different induction methods. (**A**) Wounding by axe. (**B**) Burning-chisel-drilling. (**C**) Biological inocula. (**D**) Chemical inducers.

Agarwood formation were the reactions to hurts. Resin were produced and accumulated around the wound sites. As the locations and areas of wounding sites agarwood are different in different artificial techniques, the appearances of induced agarwood varied (Figure 1 and Table 1). Wounding by an axe was tried on the trunk of trees. Agarwood covered the whole transection and are usually flakes with dark dot resins on one side (Figure 1A). The burning-chisel induced agarwood are chops with resins around the holes’ wound (Figure 1B). Non-conventional inducing methods initiated the accumulation of resin inside the tree stems along the trunks or branches. Trees were chopped and white wood were carefully removed from the resinous tissues. Agarwood were regular thin chips covered with resin strips as shown in Figure 1C,D.

Although morphology sometimes affect the commercial prices of agarwood used in sculptures or artifacts, resin contents which is scaled as ethanol extract contents are the crucial criteria in agarwood qualification system in most agarwood producing and consuming regions [26]. Resin contents partly depend on the extent of whitewood removal, especially for the artificial agarwood whose morphology is regular. Chemical constituents including chromones and sesquiterpenes in ethanol extract are contributors to the pleasant odor and medical effects of agarwood. Thus, the chemical constituents in ethanol extracts should be carefully analyzed to assess their qualities.

### 2.2. GC-MS Analysis

**Figure 2:**
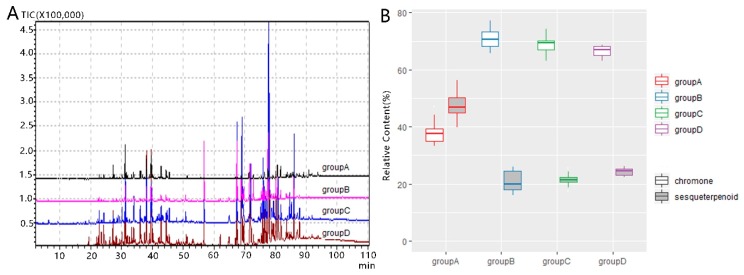
GC-MS analysis of artificial agarwood induce by different techniques. (**A**) Representative chromatographs of artificial agarwood. (**B**) Relative contents of main components detected by GC-MS. group A, wounding by axe; group B, burning-chisel-drilling; group C, chemical inducers; group D, biological inocula.

As agarwood is valued for its unique and pleasant odor, GC-MS were widely used in determining the components and access the quality. To fully acquire the chemical profiles of agarwoods, the ethanol extracts were eluted and analyzed by GC-MS. The typical TIC chromatographs of agarwood by four inducing methods are shown in Figure 2. Chemicals that showed up before retention time (RT) of 57 min were mainly sesquiterpenes and aromatic compounds. PECs were detected between 57 and 87 min with some long chain fatty acid. Steroids showed up in the last 15 min. Chemicals, except for some PECs, were identified by searching NIST14 with MS data and retention index. Tentatively identified chemicals with similarity over 80% and retention index within ±20 are listed in Table 1. Some PECs identity was done by referring to previous reports. Proportion of sesquiterpenoid was highest in groupA. PECs made up most proportions of chemicals detected in burning-chisel and non-conventional method induced agarwood (Figure 2). However, THPECs overlapped with DPECs and EPECs in GC-MS analysis (Figure 2B).

However, GC-MS is only suitable for volatile constituents and identification via GC-MS can only be achieved within limited library searching. Therefore, given the fact that PECs comprised a high percentage of the identified compounds, and while some nonvolatile PECs perhaps failed to be detected by GC-MS, UPLC-MS analysis was carried out to study the dynamic changes of PECs during the process of agarwood formation.

### 2.3. LC-MS Analysis

**Figure 3:**
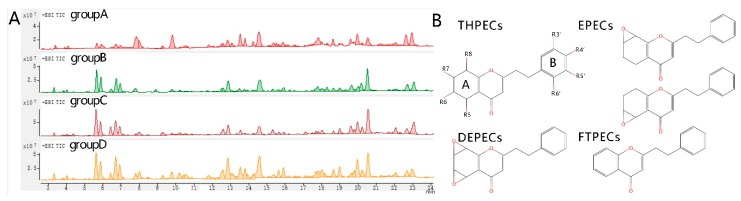
Total ion chromatograph (TIC) of agarwood produced by artificial induction methods (**A**) and the characteristic structures of four types of 2-(2-phenylethyl)chromones, (**B**) detected by LC-MS. group A, wounding by axe; group B, burning-chisel-drilling; group C, chemical inducers; group D, biological inocula.

2-(2-phenylethyl) chromones are widely reported as the characteristic constituents in agarwood in regardless of their origins and induction methods. The chromone patterns of each groups were acquired by LC-MS/MS. Total ion chromatograph (TIC) obtained by scan mode showed that all artificial agarwood were qualitatively similar (Figure 3A). Basal peaks were chosen and further identified by LC-MS/MS by production ion mode. All four types of 2-(2-phenylethyl) chromones were detected in artificial agarwood and their backbone structures are shown in Figure 3B.

**Figure 4:**
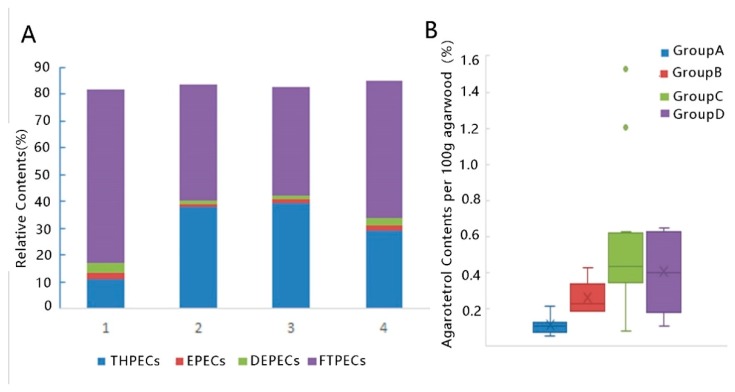
Contents of PECs in artificial agarwood groups. (**A**) Percentiles of four types of PECs in four artificial agarwood group. (**B**) Contents of agarotetrol (g/100 g) in artificial agarwood groups. group A, wounding by axe; group B, burning-chisel-drilling; group C, chemical inducers; group D, biological inocula.

Agarotetrol, 2-[2-(4-methoxphenyl)ethyl] chromone and 2-(2-phenylethly) chromone were identified by comparing their retention times and MS spectra with reference compounds. Tentative identification of chromones according to MS/MS fragments and previous reports are listed in Table 1. THPEC with 4 OHs at ring A were eluted firstly before 7 min. THPECs with less than 4 OHs, EPECs and DEPECs appear between 7 and 13 min. Chemicals detected after 13 min were molecular with least polarity such as FTPECs and sesquiterpenoids (Figure 3 and Table 2). Proportions of each type of chromone in artificial agarwood samples and the most abundant chemical was shown in Figure 4. FTPECs comprise most part of chromones in artificial agarwood (Figure 4). THPECs is lower while EPECs and DEPECS were more abundant in group A than in other groups (Figure 4). The above results strongly suggested chromones in artificial agarwood differ between groups.

### 2.4. Multivariate Analysis

**Figure 5:**
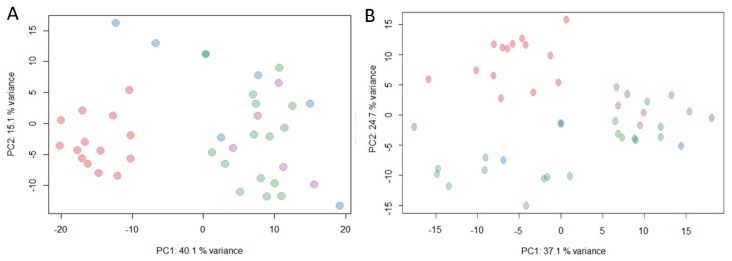
PCA based on (**A**) GC-MS and (**B**) LC-MS. Red, group A; blue, group B; green, group C; purple, group D.

As the above results show, chemical constituents in artificial agarwood are complex. To further investigate the differences within groups, all MS data were further processed for multivariate analysis. PCA respectively based on GC-MS and LC-MS showed similar grouping tendency. Most samples of group A were allocated together while samples for other groups mixed up (Figure 5A,B). Samples from group B (blue dots) scattered which suggested great variety both in sesquiterpenes and PECs.

**Figure 6:**
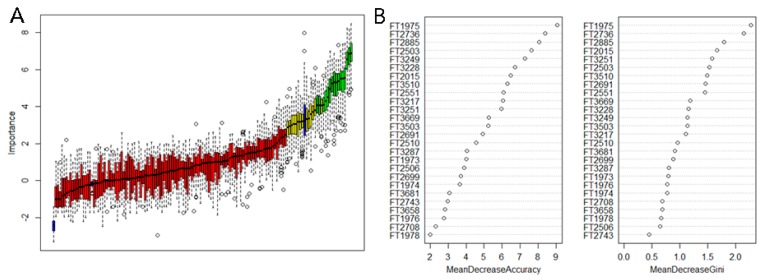
Random forest model based on LC-MS data (**A**) Feature selection based on variables used in PCA. Green, attributes confirmed important; yellow, tentative attributes; red, attributes confirmed unimportant. (**B**) VIP of factors contributing to artificial agarwood grouping in Random Forest model based on attributes selected in A.

To further identify the molecules contributing to the classification of artificial agarwood, LC-MS data were used for features selection for each group. 26 chemicals were confirmed important for artificial agarwood grouping. Random Forest based on the intensity of above 26 chemicals showed that 9 with VIP > 1.5 (Figure 6B). Those molecules were further identified by LC-MS/MS and the possible formula were listed in Table 3

Nearly all chemicals contributing to artificial agarwood classification came out at RT 14–19 min, which are most tentatively identified as FTPECs or sesquiterpenes with less polarity (Table 3).

## 3. Discussion

In this study, morphology and chemical profile of artificial agarwood by four induction methods were investigated. Artificial agarwood from different induction methods vary and results acquired from multivariate analysis strongly suggested that inducing methods affect the chemical constituents of artificial agarwood. There are over 200 chemicals found in agarwood and the number is increasingly growing. In this study, GC-MS tentatively identified 71 and LC-MS/MS tentatively identified 43 chemicals in artificial agarwood (Table 1 and Table 2). All samples had similar chemicals in the ethanol extracts while the relative contents differed (Figure 2 and Figure 4).

Unlike PECs in agarwood, although numerous sesquiterpenoids were tentatively identified in GC-MS (Table 1), no characteristic chemicals were found even in group A. One reason for relative low proportion of sesquiterpenoids in all samples is that the variety of sesquiterpenoid skeletons made them difficult to be identified without standard chemicals. Samples from group A were obviously different from other samples for its higher contents of sesquiterpenoids and lower contents of THEPCs (Figure 2 and Figure 4). One explanation for the relative lower proportions of sesquiterpenoids might be the shorter formation time in group B–D compared with group A. Sesquiterpenoids were reported be produced later than PECs [15]. THPECs are reported appear early during agarwood formation and evolved into FPECs as time goes on [11]. The formation time of samples in group A is usually over 18 months. Many samples in group B–D are about 9–16 months if known. Time length of agarwood formation might be a crucial contributor for the differences between group A and other groups. However, other factors might also contribute to the chemical composition differences. As reports on mechanisms of agarwood formation suggest differences reside between the wounding by an axe and chemical inducer method through transcriptome and microtome analysis [2,27,28]. Whether those divergences accounts for the chemical variety of artificial agarwood should be an interesting question to be resolved.

Previous studies focused on agarwood qualifications proposed certain sesquiterpenes and PECs were accumulated in high-quality agarwood. In this study, agarwood in group A contained more sesquiterpenes and PECs than other groups (Figure 2 and Figure 4), which suggested that agarwood in group A might be superior than other groups. Although many of the chemicals identified in this study can also been found in other plants, the complexity and variety of chemicals in agarwood confer its irreplaceable odor and pharmaceutical effects. Results from random forest point out several chemicals strongly correlated with samples groupings (Figure 6). It is still hard to conclude that those molecules are quality determinants. Further studies combining both chemicals analysis and medical effects or fragrant assays might help to identify the qualification markers.

Agarwood formation is believed to be the co-production of microbe and parenchyma cell at the injured sites as reported in recent research. Micro-environment of agarwood formation in conventional methods induced agarwood and the non-conventional ones differs, which can be observed by the morphology of sample in Figure 1A–D. Samples from group A were exposed to air and sunlight in whole inducing procedure while samples in group C–D were in dark and anaerobic niche until harvest. Agarwood in group B was produced in both niches; as shown in Figure 1B, holes were opened to the outside and the part near the hole were covered inside trunks. Group allocations by PCA on chemical profiles also reflect the tendency (Figure 5). Group B is closer to group A than groups C and D (Figure 5 and Figure 6). Samples in group C and group D were similar in morphology and chemical profiles (Figure 1 and Figure 6). The results strongly suggested that inducing methods affect the chemicals in cultivated agarwood. Studies focused on the microtomic and biological activities in agarwood formation under different niches might shed more light on the complex mechanisms of agarwood formation.

Agarwood is precious due to the rare formation under natural environment. Various artificial inducing techniques have been developed. In this study, cultivated agarwood induced by four methods were analyzed and compared. Types of components in the ethanol extracts are similar while differing in proportions, which strongly suggest that inducing method affect chemicals in resins of agarwood. The results are helpful for study of mechanism behind agarwood formation. Agarwood induced by wounding of axes contains more sesquiterpenes and FPECs compared with other groups. The results provide a comprehensive assessment of agarwood induced by four popular artificial methods which will help to evaluate artificial agarwood quality.

## 4. Materials and Methods

### 4.1. Agarwood Materials and Reagents

All agarwood were from plantations in Guangdong, Guangxi, Yunnan, and Hainan provinces in South China. In total, 48 artificial agarwood induced by wounding using an axe (14), burning-chisel-drilling (9), chemical inducers (17) and biological inoculation (8) were investigated. Detailed sample information was listed in Table 4. The alkaline standard (D-2887 Calibration Solution, J&K Scientific, Beijing, China) was used in GC-MS. LC-MS grade acetonitrile (Merck, Darmstadt, Germany), formic acid (TCI, Japan, Shanghai). Ultra-high purity water prepared by filtration using a Milli-Q system (Millipore, Milford, MA, USA) were used as solvents for LC-MS. Lab grade ethanol and ethyl acetate were purchased from Sigma-Aldrich (St. Louis, MO, USA). Agaroterol (111980-201602) and reference agarwood (121222-201203) were purchased from National Institutes for Food and Drug Control of China (Beijing, China). 2-(2-phenylethyl)chromone and 2-[2-(4-Methoxyphenyl)ethyl] chromone were generously gifted by Dr. Dai Haofu. Before the study, all samples were analyzed according the instructions of monography “agarwood” in Chinese Pharmacopoeia (2015 edition) to make sure the fake or adulterated samples were excluded from the following study [29].

### 4.2. Sample Preparation

All samples were ground into fine powder using a grinder. The 95/5 (*v*/*v*) ethanol/water extraction were acquired by thermal reflux. Supernatant were filtered through 0.45 um filters and stored at 4 °C before LC-MS analysis. 10 mL ethanol extracts were vaporized at 50 °C water bathing and re-dissolved in ethyl acetate and used for GC-MS analysis after desiccation and filtration.

### 4.3. GC-MS Analysis

GC-MS were performed using a GC coupled with quad-mass spectrometer (GC2010, Shimadzu, Kyoto, Japan). Analytic conditions were as follows: detector temperature, 230 °C; injection port temperature, 250 °C; column DB-5HT (30 m × 0.25 mm, Agilent Technologies AG, Waldbronn, Germany). Sample size: 1μL. The split ratio was 1:20, with helium as the carrier gas at a flow rate of 0.5 mL/min. The oven temperature program was as follows: 90 °C for 1 min; 90 °C to 150 °C with a gradient of 2 °C/min and held at 150 °C for 5 min; 150 °C to 280 °C with a gradient of 2 °C/min and held at 280 °C for 10 min. The TIC was acquired in full scanning mode (mass range 50–500 *m*/*z*). A solvent delay of 2.5 min was used. The alkaline mixture standard (C9–C40) were analyzed by the same program in GC-MS. The identification of components were assigned by the software GC-MS Postrun analysis (Shimadzu, Kyoto, Japan) by searching MS data and retention indices in National Institute of Standards and Technology (NIST, Washington DC, USA) and referring to previous reports [30,31]. Areas were recorded for all detectable peaks. Areas of each identified peaks were quantified and their proportions were calculated by GC-MS Postrun analysis (Shimadzu, Kyoto, Japan). Proportions of total sesquiterpenoid, PECs and other chemicals were separately calculated by sum of proportions of chemicals belonging to sesquiterpenoid, PECs and other chemicals.

### 4.4. LC-MS/MS Analysis

LC-MS were performed using an ultra-high performance liquid chromatography (UPLC) system (1290 Infinity II, Agilent Technologies, Singapore) coupled with triple quad-mass spectrometer (6420 Triple Quad, Agilent Technologies, Singapore). Data acquisition and LC-MS data analysis were carried out by MassHunter Acquisition^®^, MassHunter Qualitative Analysis^®^ (8.00, Agilent Technologies, Santa Clara, CA, USA). A C18 column (100 × 2.1 mm, 1.7 μm, Phenomenex, Washington, USA) was used for chromatographic separations. The mobile phases were acetonitrile (A) and 0.1% formic acid-water (B). A gradient elution was used: 10–20% at 0–5 min, 20–25% at 5–9 min, 25–30% at 9–12 min, 30–35% at 12–16 min, 35% at 16–16.3 min, 35–42% at 16.3–19 min, 42–60% at 19–28 min, 60–95% at 28–29 min, 95–10% at 29–30 min. The flow rate was0.3 mL/min and the injection volume was0.5 uL. The vaporizer gas was N_2_ at 350 ℃ and 60 psi. Positive ions were analyzed under scan, product ion, and multi reaction monitoring (MRM) model separately. In scan model, *m*/*z* ranged at 100–600 with fragmentor at 135 v. Collision energy (CE) for each precursor ions were optimized according the abundance of characteristic product ions. The molecular formula and possible structure were deduced according the precursor ion and product ions *m*/*z* according to the standards and previous research [9,32]. Areas of each identified peaks were quantified and their proportions were calculated by MassHunter Qualitative Analysis^®^. Proportions of total THEPECs, EPECs, DEPECS, and FPECs were separately calculated by sum of proportions of chemicals identified to THEPECs, EPECs, DEPECS, and FPECs.

### 4.5. Data Processing and Analysis

Raw LC-MS data were converted tomzXML format using MSconvert tools (Version 3,64 bit, proteowizard, Palo Alto, CA, USA). Raw GC-MS data were converted to mzXML format using GC-MS Postrun analysis (Shimadzu, Kyoto, Japan). Preprocessing of MS data including peak picking, peak grouping, retention time (RT) correction, and integration was performed using the XCMS implemented with R software (Version 3.5, University of Auckland, New Zealand). Each ion was identified by the RT and *m*/*z* data. Intensities of each peak were recorded and a three-dimensional matrix containing arbitrarily assigned peak indices and ion intensity information was generated. The intensities of each ions identified were normalized and the quantitative data were analyzed by several unsupervised methods and supervised methods in R. PCA (principle components analysis) was used for multivariate exploration of clusters and trends among the observation. Feature selection were performed by Boruta package in R.

## Figures and Tables

**Table 1:** Tentative identification of chemical constituents in artificial agarwood.

No	RT	Similarity	RIE	RIL	Compound Name	MW	Formula	Types
1	2.317	88	990	982	Benzaldehyde	106	C_7_H_6_O	Aromatic compound
2	4.542	88	1156	1160	Benzocyclohexane	106	C_10_H_12_	Aromatic compound
3	6.275	90	1230	1228	benzylacetone	148	C_10_H_20_O	Aromatic compound
4	19.336	84	1460	1454	7-epi-trans-seqsquisablinene hydrate	222	C_15_H_26_O	Sesquiterpenoid
5	21.000	93	1594	/	Epi-gamma-Eudesmol	222	C_15_H_26_O	Eudesmane-type Sesquiterpenoid
6	21.912	95	1612	1598	Agarospirol	222	C_15_H_26_O	Agarospirane-type Sesquiterpenoid
7	22.051	80	1615	1598	Hinesol	222	C_15_H_26_O	Guaiane-type Sesquiterpenoid
8	22.273	82	1619	1614	Guaiol	222	C_15_H_26_O	Guaiane-type Sesquiterpenoid
9	22.492	86	1624	/	Tran-guaienol	222	C_15_H_26_O	Guaiane-type Sesquiterpenoid
10	22.701	91	1628	1614	Guaiol	222	C_15_H_26_O	Guaiane-type Sesquiterpenoid
11	23.334	78	1641	1635	Isolongifolol	222	C_15_H_26_O	Sesquiterpenoid
12	23.983	75	1653	1638	Zingerone	194	C_11_H_14_O_3_	Other
13	24.202	75	1658	1645	6-isopropenyl-4,8a-dimethyl-4a,5,6,7,8,8a-heahydro-1*H*-naphthalenone	218	C_15_H_22_O	Eudesmane-type sesquiterpenoid
14	24.616	77	1669	1645	(5*s*,7*s*,10*s*)-selina-3,11-dien-9-one	218	C_15_H_22_O	Eudesmane-type sesquiterpenoid
15	26.158	80	1698	1681	2-(4a,8-Dimethyl-1,2,3,4a,5,6,7-octahydro-naphthalenenol	220	C_15_H_24_O	Eudesmane-type sesquiterpenoid
16	26.541	80	1709	1645	(5*s*,7*s*,9*s*,10*s*)-(+)-selina-3,11-dein-9-ol	222	C_15_H_26_O	Guaiane-type Sesquiterpenoid
17	27.626	80	1719	1651	Alpha endesm-11-en-1-alpha-ol	222	C_15_H_26_O	Eudesmane-type sesquiterpenoid
18	27.939	81	1743	/	Aromadendrane-4,10-diol	218	C_15_H_26_O_2_	Sesquiterpenoid
19	29.681	88	1761	/	cryptomeridiol	240	C_15_H_28_O_2_	Sesquiterpenoid
20	30.503	80	1771	/	2,6-Dimethyl-6-(4methyl-3-pentenyl)-2-cyclohexene-1-carboxaledehyde	220	C_15_H_24_O	Aromatic compound
21	31.181	80	1790	/	Kessane	220	C_15_H_24_O	sesquiterpenoid
22	31.225	80	1800	/	naphthalenone	218	C_15_H_24_O	Eudesmane-type sesquiterpenoid
22	31.499	75	1804	/	Kessanyl acetate	280	C_17_H_28_O_3_	sesquiterpenoid
23	31.913	76	1811	/	Curcumenol	234	C_15_H_22_O_2_	sesquiterpenoid
24	36.743	78	1892	/	Benzocycloheptenol	222	C_15_H_26_O	sesquiterpenoid
25	38.025	75	1913	1904	6-[1-(Hydroxymethyl)vinyl]-4,8a-dimethyl-1,2-4a-5,6,7,8a-octahydro-2-naphthalenol	236	C_15_H_24_O _2_	sesquiterpenoid
26	38.522	80	1921	1916	naphthalenone	234	C_15_H_22_O_2_	sesquiterpenoid
27	39.641	80	1938	/	phenanthrenone	250	C_15_H_22_O_3_	other
28	42.840	82	1992	/	Isopimaral	286	C_20_H_30_O	diterpenes
29	56.821	96	2272	/	2-(2-phenethyl)chromone	250	C_17_H_14_O_2_	chromones
30	59.365	/	2356	/	PEC: A ring:1OH	266	C_17_H_14_O_3_	chromones
31	62.061	/	2389	/	PEC: A ring:1OH	266	C_17_H_14_O_3_	chromones
32	64.721	/	2458	/	6,8-Dihydroxy-2-(2-phenylethyl)chromone	282	C_17_H_14_O_4_	chromones
33	64.817	/	2463	/	PEC: A ring:1OH	266	C_17_H_14_O_3_	chromones
34	64.882	/	2470	/	PEC: B ring: 1OH, 1OCH_3_	296	C_18_H_16_O_4_	chromones
35	67.430	/	2532	/	PEC: A ring: 1 OCH_3_	280	C_18_H_16_O_3_	chromones
36	67.533	94	2539	/	2-(4-Methoxyphenethyl) 4H-chromone	280	C_18_H_16_O_3_	chromones
37	68.779	/	2569	/	6,8-Dihydroxy-2-(2-phenylethyl)chromone	282	C_17_H_14_O_4_	chromones
38	69.021	/	2572	/	6,8-Dihydroxy-2-(2-phenylethyl)chromone	282	C_17_H_14_O_4_	chromones
39	69.149	/	2579	/	6-Hydroxy-8-chloro-2-(2-phenylethyl)chromone	300	C_17_H_13_ClO_3_	chromones
40	69.350	/	2583	/	PEC: A ring: 1OH, 1OCH_3_	296	C_18_H_16_O_4_	chromones
41	71.133		2633		Agaretetrol	318	C_17_H_18_O_6_	chromones
42	71.355	/	2639	/	PEC: A ring: 1OH,	266	C_17_H_14_O_3_	chromones
43	72.773	/	2678	/	EPEC: B ring: 1OH	284	C_17_H_16_O_4_	chromones
44	72.873	/	2683	/	PEC: B ring: 1OH, 1 OCH_3_	296	C_18_H_16_O_4_	chromones
45	74.124	/	2716	/	PEC: A ring: 1OH, 1 OCH_3_, B ring: 1 OCH_3_	326	C_19_H_18_O_5_	chromones
46	74.852	/	2734	/	5,8-Dihydroxy-2-[2-(4-methoxyphenyl)ethyl]chromone	312	C_18_H_16_O_5_	chromones
47	75.450	/	2751	/	EPEC: A ring: 1OH,1 OCH_3_, 1 epo	314	C_18_H_18_O_5_	chromones
48	76.361	/	2780	/	EPEC: A ring: 2OH, 1 epo	300	C_17_H_16_O_5_	chromones
49	77.476	/	2813	/	PEC:B ring: 2 OCH_3_	310	C_19_H_18_O_4_	chromones
50	77.678	/	2819	/	PEC: A ring: 2OH, B ring: 1 OCH_3_	312	C_18_H_16_O_5_	chromones
51	79.350	/	2863	/	PEC: A ring: 1OH, 1 OCH_3_	296	C_18_H_16_O_4_	chromones
52	80.050	/	2890	/	Stigmasterol	412	C_29_H_4_8O	stigmasterol
53	80.208	/	2901	/	THPEC: A ring: 4OH B ring 1 OCH_3_	348	C_18_H_20_O_7_	chromones
54	80.616	/	2907	/	PEC: A ring: 1OH, B ring: 1OCH3	296	C_18_H_16_O_4_	chromones
55	81.142	/	2923	/	PEC: A ring:2OH, B ring: 1OCH3, 1OH	328	C_18_H_16_O_6_	chromones
56	81.676	/	2939	/	PEC: A ring: 1 OCH_3_, B ring: 1 OCH_3_, 1OH	326	C_19_H_18_O_5_	chromones
57	83.152	/	2985	/	PEC: A ring: 1 OCH_3_, 1OH; B ring: 1 OCH_3_, 1OH	342	C_19_H_18_O_6_	chromones
58	83.484	/	2995	/	PEC: A ring: 1 OCH_3_, B ring: 1 OCH_3_, 1OH	326	C_19_H_18_O_5_	chromones
59	83.821	/	3006	/	PEC: A ring:2OH, B ring: 1 OCH_3_, 1OH	328	C_18_H_16_O_6_	chromones
60	84.961	/	3042		PEC: A ring: 1 OCH_3_, 2OH; B ring: 1 OCH_3_,	342	C_19_H_18_O_6_	chromones
61	85.498	/	3059		PEC: A ring: 1OH, B ring: 1 OCH_3_, 1OH	312	C_18_H_16_O_5_	chromones
62	85.916	/	3073	/	PECs: A ring: 2 OCH_3_,B: 1 OCH_3_	340	C_20_H_20_O_5_	chromones
63	86.952	/	3106	/	PEC: A ring: 1OH, B ring: 1 OCH_3_, 1OH	312	C_18_H_16_O_5_	chromones
64	87.695	/	3130	/	PEC: A ring: 1 OCH_3_, 1OH; B ring: 1OCH3	326	C_19_H_18_O_5_	chromones
65	88.537	88	3178	/	stigmasterol	412	C_29_H_48_O	Steriods
66	89.950	/	3205	/	PEC: A ring: 1 OCH_3_, 1OH; B ring: 1 OCH_3_, 1OH	342	C_19_H_18_O_6_	chromones
67	90.813	92	3234	/	gamma-Sitosterol	414	C_29_H_50_O	Steriods
68	91.042	/	3242	/	PEC: A ring: 1Cl, 1OH; B ring: 1 OCH_3_, 1OH	346	C_18_H_15_ClO_5_	chromones
69	91.814	/	3268	/	PEC: A ring: 2 OCH_3_; B ring: 1 OCH_3_, 1OH	356	C_20_H_20_O_6_	chromones
70	93.730	/	3334	/	PEC: A ring: 1 OCH_3_, 1OH; B ring: 1 OCH_3_, 1OH	342	C_19_H_18_O_6_	chromones
71	94.495	87	3361	/	Gamma-Sitostenone	412	C_29_H_48_O	Steriods

“/” not determined, PEC, 2-(2-phenylethyl)chromone; THPECs, tetrahydro-2-(2-phenylethyl) chromones; EPECs, epoxy-(2-phenylethyl)chromones; diepoxy-(2-phenylethyl)chromones (DEPECs).

**Table 2:** Tentative identification of 2-(2-phenylethyl) chromones (PECs) in artificial agarwood.

No	RT (min)	MF	MW	Compounds	Fragment Ions
1	2.628	C_19_H_23_O_9_	394	THPECs: A ring:4 OH, B ring:1OH,2 OCH_3_	377,361,331,167
2	3.328	C_18_H_21_O_8_	364	THPECs: A ring:4 OH, B ring:1OH,1 OCH_3_	347, 329, 301, 137
3	3.85	C_18_H_21_O_8_	364	THPECs: A ring:4 OH, B ring:1OH,1 OCH_3_	347, 329, 301, 137
4	4.028	C_18_H_21_O_8_	364	THPECs: A ring:4 OH, B ring:1OH,1 OCH_3_	347,329,301,137
5	5.616	C_17_H_18_O_6_	318	Agarotetrol *	301, 283, 255, 91
6	5.876	C_18_H_20_O_7_	348	THPECs: A ring:4 OH, B ring:1 OCH_3_	331,313,121
7	6.4	C_17_H_18_O6	318	THPECs: A ring:4 OH,	301, 283, 255, 91
8	6.689	C_17_H_18_O_6_	318	THPECs: A ring:4 OH,	301, 283, 255, 91
9	6.922	C_18_H_20_O_7_	348	THPECs: A ring:4 OH, B ring:1 OCH_3_	331,313,121
10	7.788	C_18_H_18_O_7_	346	EPECs: A ring:2 OH, B ring:1OH, 1 OCH_3_	329, 301, 137
11	7.988	C_18_H_18_O_7_	346	EPECs: A ring:2OH, B ring:1OH, 1 OCH_3_	329, 301, 137
12	7.988	C_18_H_18_O_7_	358	DEPECs: B ring 1 OH, 2 OCH_3_	329, 167
13	8.427	C_18_H_18_6__O	330	EPECs: A ring:2 OH, B ring:1 OCH_3_	311,121
14	8.992	C_18_H_16_O_6_	328	DEPECs: B ring 1 OH, 1 OCH_3_	137
15	9.261	C_19_H_22_O_7_Cl	396	THPECs: A ring:3OH,1Cl OCH_3_, B ring:1 OCH_3_	379, 121
16	9.772	C_18_H_18_6__O	328	DEPECs: B ring 1 OH, 1 OCH_3_	301,137
17	10.238	C_18_H_18_6__O	332	THPECs: A ring:3 OH, B ring:1 OCH_3_	315,121
18	10.533	C_19_H_22_O_5_	330	THEPECs: A ring:2 OH, B ring:1 OCH_3_	313,121
19	11.783	C_18_H_18_6__O	328	DEPECs: B ring:1 OCH_3_,1OH	301,137
20	12.505	C_18_H_20_ClO_6_	366	THPECs: A ring:3OH,1Cl OCH_3_, B ring:1 OCH_3_	349, 121
21	12.583	C_17_H_18_ClO_5_	336	THPECs: A ring:1Cl, 3 OH	319, 301, 283, 273, 91
22	12.849	C_19_H_22_O_5_	330	THPECs: A ring:3 OH, B ring:1 OCH_3_	313,121
23	13.083	C_19_H_16_O_6_	342	FTPECs: A ring:1 OCH_3_,1OH B ring:1 OCH_3_,1OH	237,137
24	13.538	C_19_H_16_O_6_	342	FTPECs: A ring:1 OCH_3_,1OH B ring:1 OCH_3_,1OH	237,137
25	13.805	C_18_H_18_5__O	312	FTPECs: A ring:1OH, B ring:1 OCH_3_,1OH	137
26	14.244	C_18_H_16_O_5_	312	FTPECs: A ring:2OH, B ring:1 OCH_3_	121
27	14.556	C_18_H_16_O_5_	312	FTPECs: A ring: 1OH, 1OCH3 B ring:1 OH	107
28	15.105	C_19_H_1_8__O_5_	326	FTPECs: A ring: 2OH, B ring: 1OH, 1 OCH_3_	137
29	15.372	C_20_H_20_O_6_	356	FTPECs: A ring: OH, 2 OCH_3_, B ring: 1 OCH_3_	121,
30	15.899	C_20_H_20_O_6_	356	FTPECs: A ring: 2 OCH_3_, B ring: 1OH, 1 OCH_3_	137,221
31	16.188	C_17_H_14_O_3_	266	FTPECs: B ring: 1OH	107,161
32	16.533	C_18_H_16_O_4_	296	FTPECs: A ring: 1OH, 1 OCH_3_, B ring: 1 OCH_3_	121
33	17.833	C_19_H_18_O_5_	326	FTPECs: A ring: 2 OCH_3_, B ring: 1OH	107
34	17.999	C_18_H_16_O_4_	296	FTPECs: A ring: 1OH, 1 OCH_3_, B ring: 1 OCH_3_	121
35	18.310	C_19_H_18_O_5_	326	FTPECs: A ring: 1OH, 1 OCH_3_, B ring: 1 OCH_3_	121
36	18.655	C_18_H_16_O_4_	296	FTPECs: A ring: 1OH, 1 OCH_3_	91
37	19.010	C_1_7__H_1_4__O_3_	266	FTPECs: B ring: 1OH	107
38	20.232	C_20_H_20_O_5_	340	FTPECs: A ring: 2 OCH_3_, B ring: 1 OCH_3_	121
39	20.549	C_19_H_18_O_4_	310	FTPECs: A ring: 2 OCH_3_	91
40	21.688	C_18_H_16_O_3_	280	2-[2-(4-Methoxyphenyl)ethyl] chromone *	121
41	22.088	C_17_H_14_O_2_	250	2-(2-phenyethyl) chromone *	91
42	20.232	C_20_H_20_O_5_	340	FTPECs: A ring: 2 OCH_3_, B ring: 1 OCH_3_	121
43	20.549	C_19_H_18_O_4_	310	FTPECs: A ring: 2 OCH_3_	91

* Identified by reference chemicals, THEPECs, Tetrahydro-2-(2-phenylethyl)-chromones; EPECs, epoxy-(2-phenylethyl)chromones; DEPEC, diepoxy-(2-phenylethyl)chromones; FTPECs, 2-(2-phenylethyl)-chromones.

**Table 3:** Tentatively identified chemicals contributing to artificial agarwood grouping by LC-MS/MS.

No	RT	MW	Type	Molecular Formula
FT1975	14.6	334	THPECs: A ring: 4OH, B ring: 1OH	C_17_H_18_O_7_
FT2736	17.916	326	FTPECs: A ring: 2OCH_3_, B ring: 1OH	C_19_H_18_O_5_
FT2885	17.633	266	FTPECs: B ring: 1OH	C_17_H_14_O_3_
FT2015	14.7	304	Unknown	C_20_H_32_O_2_
FT3251	19.333	300	FTPECs: A ring: 1OH, 1Cl	C1_7_H_13_ClO_3_
FT2503	16.033	282	Sesquiterpenoid	C_16_H_26_O_4_
FT3510	19.85	218	Sesquiterpenoid	C_15_H_22_O
FT2691	16.567	296	FTPECs: A ring: 1OH, 1OCH_3_, B ring: 1OCH_3_	C_18_H_16_O_4_
FT2551	17.85	326	FTPECs: A ring: 2OCH_3_, B ring: 1OH	C_19_H_18_O_5_

THEPECs, Tetrahydro-2-(2-phenylethyl)-chromones; FTPECs, 2-(2-phenylethyl)-chromones.

**Table 4:** Sample information.

No	Group	Origin	No	Group	Origin	No	Group	Origin
S1	A	Zhongshan, Guangdong	S17	B	Haikou, Hainan	S33	C	Haikou, Hainan
S2	A	Zhongshan, Guangdong	S18	B	Dongguan, Guangdong	S34	C	Haikou, Hainan
S3	A	Zhongshan, Guangdong	S19	B	Dongguan, Guangdong	S35	C	Haikou, Hainan
S4	A	Dongguan, Guangdong	S20	B	Dongguan, Guangdong	S36	C	Haikou, Hainan
S5	A	Dongguan, Guangdong	S21	B	Haikou, Hainan	S37	C	Haikou, Hainan
S6	A	Dongguan, Guangdong	S22	B	Haikou, Hainan	S38	C	Haikou, Hainan
S7	A	Dongguan, Guangdong	S23	B	Haikou, Hainan	S39	C	Haikou, Hainan
S8	A	Dongguan Guangdong	S24	C	Yulin, Guangxi	S40	C	Zhongshan, Guangdong
S9	A	Dongguan, Guangdong	S25	C	Zhongshan, Guangdong	S41	D	Puer, Yunan
S10	A	Dongguan, Guangdong	S26	C	Zhongshan, Guangdong	S42	D	Puer, Yunan
S11	A	Haikou, Hainan	S27	C	Zhongshan, Guangdong	S43	D	Puer, Yunan
S12	A	Hainan, Haikou	S28	C	Zhongshan, Guangdong	S44	D	Xishuangbana Yunnan
S13	A	Haikou, Hainan	S29	C	Zhongshan, Guangdong	S45	D	Puer, Yunan
S14	A	Haikou, Hainan	S30	C	Zhongshan, Guangdong	S46	D	Zhongshan, Guangdong
S15	B	Huazhou, Guangxi	S31	C	Huazhou, Guangxi	S47	D	Haikou, Hainan
S16	B	Zhongshan, Guangdong	S32	C	Haikou, Hainan	S48	D	Beihai, Guangxi
